# Transcystic biliary tract exploration during single-stage treatment for cholecystitis/symptomatic cholelithiasis plus choledocholithiasis using the SpyGlass system: a favorable therapeutic proposal

**DOI:** 10.1093/jscr/rjaf495

**Published:** 2025-07-13

**Authors:** Máximo Vicente Torres Guaicha, Tábata Lissette Tinoco Ortiz, Santiago Borja Villacres, Francisco Troya, Santiago Andrés Muñoz-Palomeque

**Affiliations:** Faculty of Medicine, Central University of Ecuador, Quito, 170521, Ecuador; Department of Surgery, Division of General Surgery, Hospital Metropolitano, Av. Mariana de Jesús Oe 7/47, Quito,170521, Ecuador; Department of Surgery, Nueva Clínica Internacional, Fundación Obesidad Ecuador, Quito, 170508, Ecuador; Pontificia Universidad Católica del Ecuador, Quito, 170525, Ecuador; Department of Surgery, Nueva Clínica Internacional, Quito, 170508, Ecuador; Department of General Surgery, Hospital Metropolitano, Quito, 170508 Ecuador; Faculty of Medical, Health and Life Sciences, Universidad Internacional del Ecuador, Quito, 170411, Ecuador

**Keywords:** bile ducts, biliary tract diseases, cholecystectomy, choledocholithiasis, surgery

## Abstract

Choledocholithiasis is traditionally managed via a two-stage approach involving endoscopic retrograde cholangiopancreatography followed by laparoscopic cholecystectomy. However, this method carries significant morbidity and logistical challenges. We report a case of a 70-year-old woman with symptomatic choledocholithiasis and mild pancreatitis successfully treated with a single-stage laparoscopic cholecystectomy and transcystic bile duct exploration using the SpyGlass DS II system. This technique enabled direct stone visualization, electrohydraulic lithotripsy, and successful ductal clearance without the need for choledochotomy or papillary trauma. The patient experienced an uneventful recovery, resuming oral intake within 6 hours and being discharged within 36 hours postoperatively. SpyGlass-assisted transcystic exploration offers important advantages over endoscopic retrograde cholangiopancreatography, including reduced complication rates, shorter hospital stays, and cost-effectiveness in experienced centers. This case supports the integration of SpyGlass technology into surgical algorithms for choledocholithiasis management, especially in high-risk or anatomically challenging patients. Further studies are warranted to confirm its role in broader clinical practice.

## Introduction

Gallstone disease affects ~6% of the global population, with a higher prevalence among women and in certain geographic regions such as South America (up to 11%) [1]. Approximately 10%–15% of patients with cholelithiasis will eventually develop common bile duct (CBD) stones, which can lead to complications such as cholangitis and pancreatitis [2].

Traditionally, choledocholithiasis is managed in two stages: first endoscopic retrograde cholangiopancreatography (ERCP) with sphincterotomy, followed by elective laparoscopic cholecystectomy. While effective, this approach is associated with ERCP-related complications and logistical challenges, especially in cases with altered anatomy or difficult papillary access. The “Rendezvous” procedure (intraoperative ERCP during cholecystectomy) also presents coordination and procedural limitations [3].

Single-stage laparoscopic CBD exploration (LCBDE) during cholecystectomy has emerged as a viable treatment. Studies have shown this approach to be equally effective—and sometimes superior—to the sequential ERCP-plus-surgery method [4].

Technological innovations, particularly the SpyGlass™ single-operator system, have significantly advanced bile duct exploration. The SpyGlass DS II, a 3.6-mm-diameter digital cholangioscope, allows direct visualization and therapeutic intervention via the cystic duct. It accommodates instruments such as electrohydraulic and laser lithotripsy probes, extraction baskets (SpyBasket®), and biopsy forceps (SpyBite®), facilitating ductal clearance without choledochotomy and enabling immediate confirmation of stone removal [5].

Here, we present a case experience involving single-stage laparoscopic cholecystectomy with transcystic cholangioscopy and electrohydraulic lithotripsy using the SpyGlass DS II system, demonstrating cost and morbidity advantages compared to the traditional two-stage approach or conventional open CBD exploration.

## Case report

**Figure 1:**
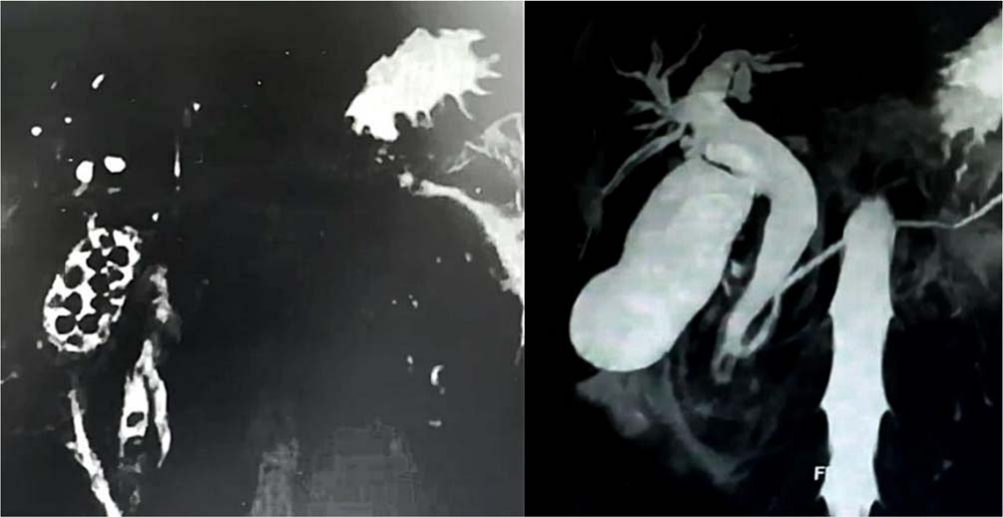
Magnetic resonance cholangiography reconstruction (360°) demonstrating the presence of multiple gallbladder stones and two embedded in the distal common bile duct.

A 70-year-old woman with a history of arterial hypertension presented with a 72-hour history of epigastric pain radiating to the back, jaundice, nausea, and vomiting. Physical examination revealed a positive Murphy's sign. Laboratory tests showed leukocytosis, elevated liver enzymes aspartate aminotransferase (AST) 17× upper limit normal, alanine transaminase (ALT) 11× upper limit normal, gamma-glutamyl transpeptidase (GGT) 18× upper limit normal, direct hyperbilirubinemia, and mild pancreatitis without organ dysfunction. Abdominal ultrasound revealed gallbladder wall thickening and a 16 mm CBD. Magnetic resonance imaging (MRI) identified two CBD stones (4.4 and 4.0 mm) (Fig. 1).

**Figure 2:**
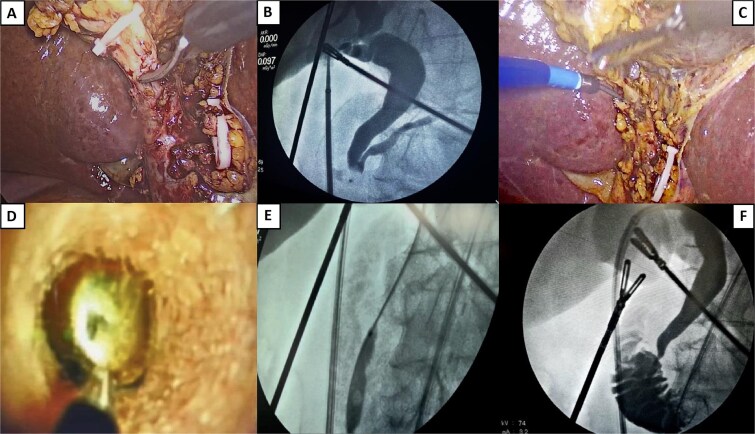
Surgical procedure. (A) Opening of the cystic duct. (B) Initial cholangiography showing no passage into the duodenum and passage into the main pancreatic duct. (C) Introduction of a transcystic SpyGlass over a hydrophilic guidewire. (D) Stone lodged in the distal common bile duct, ready for fragmentation. An electrohydraulic lithotripsy probe is visible, aimed at the center of the stone. (E) Sphincteroplasty by balloon dilation. A notch is visible, coinciding with the sphincter of the Oddi area. (F) Final cholangiography showing free passage of contrast into the duodenum, with no filling defect.

After supportive treatment for pancreatitis, the patient underwent laparoscopic cholecystectomy with transcystic CBD exploration using the SpyGlass DS II system and electrohydraulic lithotripsy (Fig. 2). The 150-minute procedure included intraoperative cholangiography and successful stone fragmentation.

Under general anesthesia and in reverse Trendelenburg position, four ports were placed in the standard cholecystectomy configuration. Intraoperative findings revealed a Parkland grade 3 gallbladder and Zühlke grade II adhesions between the gallbladder fundus and the anterior abdominal wall. Moderate perivesicular inflammatory fluid was sampled for culture. Careful dissection proceeded to achieve the critical view of safety. A long, wide cystic duct and a dilated CBD were identified. Hem-o-lock clips were placed on the cystic artery and distal cystic duct, which was then partially incised.

A Navigator 11–13F catheter was introduced through the midclavicular port for initial cholangiography, which revealed filling defects in the distal bile duct and no contrast passage into the duodenum, but with contrast reflux into the pancreatic duct. A hydrophilic guidewire was advanced into the duodenum using the Seldinger technique, followed by the insertion of the SpyGlass DS II system (Boston Scientific). This 3.5-mm-diameter, 65–cm-long scope features 30° tip angulation.

Choledochoscopy confirmed two stones occupying ~90% of the distal CBD lumen. Electrohydraulic lithotripsy was used to fragment stones delivering multiple shockwave pulses to achieve complete stone fragmentation. Then a percutaneous balloon was used for sphincteroplasty over 3 minutes, followed by saline flushing and fragmentation extraction using the SpyGlass instruments. Free contrast passage into the duodenum was confirmed, and final cholangiography showed no residual filling defects or contrast leakage, verifying biliary and duodenal integrity.

The patient had an uneventful recovery, tolerated oral intake 6 hours postoperatively, and was discharged 36 hours after surgery.

## Discussion

Prompt management of choledocholithiasis is critical to avoid complications such as cholangitis or pancreatitis [6]. Although ERCP is a well-established treatment, it carries significant risks, including pancreatitis (3%–5%, up to 10% in high-risk patients), duodenal perforation (<1%), bleeding (1%–2%), and cholangitis (<1%) [5, 7]. ERCP failure occurs in 5%–10% of cases, particularly with anatomical challenges such as periampullary diverticula [8].

Single-stage strategies like LCBDE during cholecystectomy offer several benefits, including high success rates (88%–92%), comparable morbidity to ERCP, and improved hospitalization metrics [6]. Abdelkader *et al.* [5] reported a 100% success rate in CBD stone clearance using SpyGlass compared to 89% with conventional ERCP, along with shorter hospital stays and lower complication rates.

Although the SpyGlass system entails significant equipment and training costs, the reduction in hospital stays and procedures may offset these expenses [5]. Open choledochotomy is now rarely used due to higher morbidity and invasiveness, and even laparoscopic choledochotomy involves T-tube drain placement and risk of bile leak or stricture formation [7].

The transcystic approach, particularly with SpyGlass assistance, avoids these complications. In difficult cases, a mini choledochotomy can still accommodate SpyGlass with reduced risk [7]. Notably, transcystic SpyGlass procedures avoid papillary trauma, reducing the incidence of postprocedural pancreatitis.

The clinical advantages of SpyGlass include

- direct visualization: it enables precise localization and real-time monitoring of stone fragmentation and extraction [3].
- advanced lithotripsy: electrohydraulic and laser probes achieve 90%–95% success in complex stone fragmentation under direct vision [9].
- targeted biopsy: SpyBite® forceps allow *in situ* sampling of biliary lesions with higher diagnostic accuracy than traditional blind ERCP brushings [10].
- balloon sphincteroplasty: it facilitates stone passage while preserving the sphincter of Oddi, reducing bleeding and perforation risks [11].
- lower complication rates: by avoiding endoscopic manipulation and fluoroscopy, intraoperative transcystic exploration reduces the risks of pancreatitis and infection [10, 12].

In our case, complete ductal clearance was achieved without complication, consistent with the existing literature on SpyGlass-assisted transcystic exploration. No postoperative leaks, infections, or pancreatitis occurred.

## Conclusion

SpyGlass-assisted transcystic bile duct exploration during laparoscopic cholecystectomy represents a safe, effective, and minimally invasive alternative to conventional two-stage ERCP plus surgery. This technique offers combined diagnostic and therapeutic capabilities in a single session, avoiding choledochotomy and minimizing ERCP-associated morbidity.

**Figure 3:**
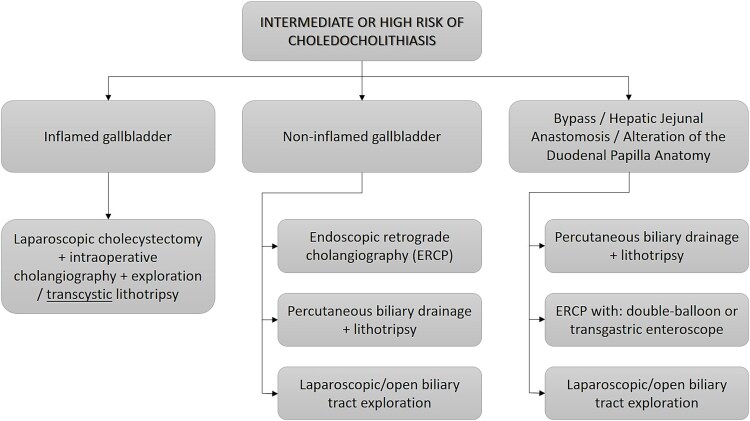
New flowchart proposed for the management of patients at intermediate or high risk of choledocholithiasis due to the advent of new technologies such as SpyGlass.

It is particularly well suited for high-risk or anatomically complex CBD stone cases, provided appropriate training and resources are available. Although further studies are needed to define its place in global clinical guidelines, current data and international algorithms increasingly support its integration into advanced biliary surgical practice (Fig. 3). Our findings further validate this approach as both clinically effective and safe.

## Data Availability

All case data are available from the lead author, Dr. Maximo Torres Guaicha (email: dr.max.torres@hotmail.com).
